# Genome-wide association for milk production and female fertility traits in Canadian dairy Holstein cattle

**DOI:** 10.1186/s12863-016-0386-1

**Published:** 2016-06-10

**Authors:** Shadi Nayeri, Mehdi Sargolzaei, Mohammed K. Abo-Ismail, Natalie May, Stephen P. Miller, Flavio Schenkel, Stephen S. Moore, Paul Stothard

**Affiliations:** Department of Agricultural, Food and Nutritional Science, University of Alberta, Edmonton, Alberta T6G 2P5 Canada; Department of Animal Bioscience, Centre for the Genetic Improvement of Livestock, University of Guelph, Ontario, Canada; The Semex Alliance, Guelph, Canada; Department of Animal and Poultry Production, Damanhour University, Damanhour, Egypt; Invermay Agricultural Centre, AgResearch Limited, Mosgiel, New Zealand; Agriculture and Food Innovation, Centre for Animal Science, University of Queensland, Queensland, Australia

**Keywords:** Genome-wide association analysis, Functional analysis, Holstein cattle, Female fertility and milk production

## Abstract

**Background:**

Genome-wide association studies (GWAS) are a powerful tool for detecting genomic regions explaining variation in phenotype. The objectives of the present study were to identify or refine the positions of genomic regions affecting milk production, milk components and fertility traits in Canadian Holstein cattle, and to use these positions to identify genes and pathways that may influence these traits.

**Result:**

Several QTL regions were detected for milk production (MILK), fat production (FAT), protein production (PROT) and fat and protein deviation (FATD, PROTD respectively). The identified QTL regions for production traits (including milk production) support previous findings and some overlap with genes with known relevant biological functions identified in earlier studies such as *DGAT1* and *CPSF1.* A significant region on chromosome 21 overlapping with the gene *FAM181A* and not previous linked to fertility in dairy cattle was identified for the calving to first service interval and days open. A functional enrichment analysis of the GWAS results yielded GO terms consistent with the specific phenotypes tested, for example GO terms GO:0007595 (lactation) and GO:0043627 (response to estrogen) for milk production (MILK), GO:0051057 (positive regulation of small GTPase mediated signal transduction) for fat production (FAT), GO:0040019 (positive regulation of embryonic development) for first service to calving interval (CTFS) and GO:0043268 (positive regulation of potassium ion transport) for days open (DO). In other cases the connection between the enriched GO terms and the traits were less clear, for example GO:0003279 (cardiac septum development) for FAT and GO:0030903 (notochord development) for DO trait.

**Conclusion:**

The chromosomal regions and enriched pathways identified in this study confirm several previous findings and highlight new regions and pathways that may contribute to variation in production or fertility traits in dairy cattle.

**Electronic supplementary material:**

The online version of this article (doi:10.1186/s12863-016-0386-1) contains supplementary material, which is available to authorized users.

## Background

Milk production and fertility are two economically important traits affecting profitability in dairy cattle. These traits are polygenic, affected by many genes and variants, each with small effects on the observed phenotype [1]. Improvements in management and nutrition, along with intense genetic selection have increased milk production in recent decades. However, selection has also changed the reproductive physiology of the cow and led to a decrease in reproductive efficiency [2]. For example, time in estrus has been reduced to less than 8 hours in lactating dairy cows [3], pregnancy rate has decreased, days open and services per conception has increased [2]. In the last decade, advances in genome sequencing technologies and availability of a tremendous number of genetic variants in the form of single nucleotide polymorphisms (SNP) (Bovine HapMap Consortium, 2009) have led to the application of genomic selection (GS) [4]. Genomic selection is based on linkage disequilibrium (LD) of unknown functional mutations and SNP genotypes that are spread out across the whole genome [4]. Incorporation of functional mutations into genotyping panels could increase GS accuracy and applicability across populations [1, 5].

Many quantitative regions and candidate genes associated with milk production and fertility have been identified by means of genome-wide association analysis studies (GWAS) [6–8]. Several QTL regions and genes associated with milk yield, fat yield, protein yield, fat deviation and protein deviation have been reported in previous studies [9–12]. Earlier studies have also identified strong functional candidate genes that affect milk production traits such as *DGAT1* and *GHR* [13, 14]. Similarly, important genomic associations for fertility traits were found in previous GWAS studies, including significant QTLs for calving to first service interval [15–17], days open [18], cow non-return rate [15, 19], heifer non-return rate [20], daughter pregnancy rate [12, 21], age at puberty [22] and interval from first service to last service for cows and heifers [15].

Most of the previously described genetic variants, however, are not causal, but rather are in linkage disequilibrium with the functional mutation. The level of LD is a limiting factor for the precision of QTL location detection in dairy cattle populations [23]. This is because even SNPs at long distances from the QTL may show associations with the phenotypic trait of interest due to extended LD [24]. Identifying pathways and genes that are associated with significant SNPs can give us a deeper biological insight into expression mechanisms of the trait under study [1, 25]. Refining the position of QTL regions harbouring candidate genes, and identifying causal mutations underlying variation in complex traits, can lead to an increase in accuracy of selection for these traits [26].

The goals of the current study were to identify or refine the position of QTL regions for milk production (MILK), fat production (FAT), protein production (PROT), fat deviation (FATD), protein deviation (PROTD), heifer first service to calving interval (FSTCh), calving to first service interval (CTFS), daughter fertility (DF), and days open (DO) in Canadian Holstein dairy cattle. Additionally we performed an enrichment analysis to test for overrepresentation of significant SNPs in biological pathways.

## Results and discussion

### Association analysis

**Fig. 1:**
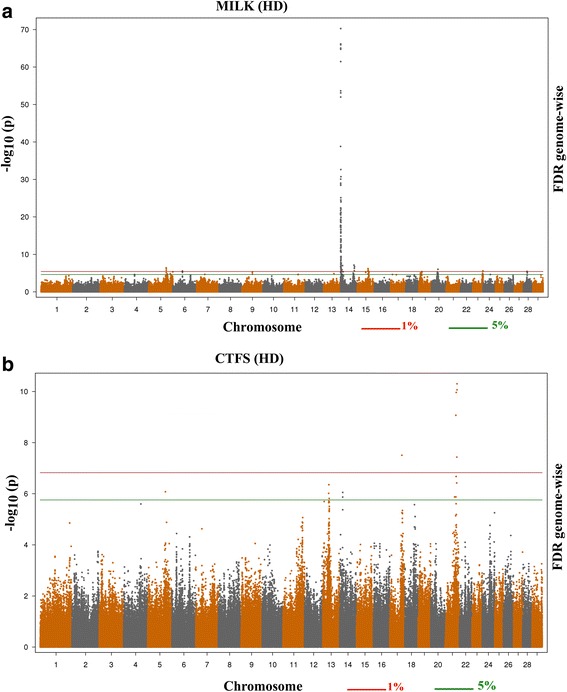
The –log_10_ of the P-values for association with SNPs is plotted for **a**. milk production (MILK) and **b**. calving to first service intervals (CTFS) in Holstein dairy cattle. Chromosome number is indicated on the horizontal axis. The red line is the threshold for significant SNPs at 1 % FDR. The green line is the threshold for significant SNPs at 5 % FDR

Association analysis identified strong associations for most of the production traits (Additional file 1: Figure S1 A-D) and some of the fertility traits (Additional file 2: Figure S2 A-C) in this study. Representative Manhattan plots are shown in Fig. 1, for milk production (MILK) and calving to first service interval (CTFS) traits. No significant associations were detected for FSTCh and DF. For those traits yielding significant associations, the number of significant SNPs identified at a genome-wise FDR of 5 % varied from 1,416 for PROTD to 8 for DO (Table 1). Q-Q plots comparing the observed distribution of –log (P-value) to the expectation under null hypothesis are shown in Additional file 1: Figure S1 E-I (milk production traits) and Additional file 2: Figure S2 D-G (fertility traits). The plots show a distribution close to the expected distribution line for CTFS (λ_median_ = 1.0567), DO (λ_median_ = 1.0115) and some production traits (MILK λ_median_ = 1.0056; FAT λ_median_ = 0.9558; PROT λ_median_ = 1.05), whereas there were strong deviations from expectation for FATD (λ_median_ = 0.7844) and PROTD (λ_median_ = 0.8969). When a high-density marker panel is used in single maker association analysis potentially a large number of markers in linkage disequilibrium may display association (and similar low P-values) with the same QTL region. This yields many significant tests that are not independent and, therefore, deviate from the expected distribution of test statistics. However, this does not imply in an overall inflation of P-values. The deviations from expectations observed for milk, fat, protein and fat deviations appear to be due largely to the strong effect of the *DGAT1* gene and the many SNPs in linkage disequilibrium that show some degree of association with it. When BTA14 was excluded from the analyses to assess this possibility, the λ_median_ values for milk production (MILK), fat production (FAT), fat deviation (FATD) and protein deviation (PROTD) were 1.0605, 1.0896, 1.1005 and 0.9709, respectively, much closer to the expectation.

**Table 1:** The number of significant SNPs (5 % and 1 % genome-wise FDR) using single SNP regression mixed linear model on imputed BovineHD (777 k) genotypes in Holstein dairy cattle

Trait	Heritability	No. of sig SNPs (1 % FDR)	No. of sig. SNPs (5 % FDR)
Milk production	0.410	221	292
Fat production	0.340	595	813
Protein production	0.370	41	87
Fat deviation (%)	0.370	998	1,230
Protein deviation (%)	0.370	912	1,416
Daughter fertility	0.070	0	0
First service to calving interval (heifer)	0.033	0	0
Calving to first service interval	0.072	8	20
Days open	0.102	8	0

### Production traits

#### Milk production (MILK)

In total 292 SNPs were found to be significant for milk production at a genome-wise FDR of 5 % (Additional file 3: Table S1). Highly significant SNPs (genome-wise 1 % FDR) were mostly localized on BTA 5, 6, 14, 15, 20 (Fig. 1a).

Many of the strong associations detected in this study support previously reported regions for the same or correlated traits. For example, a region containing numerous highly significant SNPs on BTA14 includes *DGAT1*, a gene with a major effect on milk fat content [13] and several other production traits [6, 11, 27–29]. A peak on BTA20 overlaps with growth hormone receptor (*GHR*) [14, 30, 31] and a nearby peak at 31–32 Mb contains several genes previously identified as potentially influencing milk production, milk fat percentage, lean meat yield, carcass weight, residual feed intake, age at puberty and male fertility traits in beef cattle (Additional file 4: Table S3) [32–36]. We have also detected regions that were previously identified on BTA5 at position 87 and 107 Mb for milk yield [37]. The presence of a QTL at confidence interval of 45–52 Mb on BTA6 was confirmed by previously reported significant QTLs found for clinical mastitis in Norwegian Red dairy cattle [38]. A significant peak on BTA15 at the genomic interval of 54 to 58 Mb in this study is close to a significant SNP that was reported to be associated with persistency of milk production in dairy cattle by Kolbehdari et al. [9], which is located in an intronic region of the *CD44* gene.

The most significant SNP (BovineHD1400000216: rs134432442) for this trait was located within the *CPSF1* gene on BTA14. Cochran et al. [39] has reported this SNP to be significantly associated with fat yield and fat percentage in Holstein cattle. Other highly significant (genome-wise FDR ≤ 1 %) SNPs in this study are located within genes *TONSL* (BovineHD1400000206: rs137472016), *CYHR1* (BovineHD1400000204: rs137727465), *FOXH1* and *PPP1R16A* (ARS-BFGL-NGS-57820: rs109146371) (Additional file 3: Table S1).

Of the 7,586 SNPs that were introduced as candidate SNPs (P-value < 0.01) to SNP2GO for enrichment analysis, a total of 1,576 were enriched in biological pathways that may provide better insight into key pathways and genes associated with milk production. Enrichment analysis found 545 significant GO terms (genome-wise FDR ≤ 1 %) with a minimum of 10 genes associated with each GO term (Additional file 5: Table S5). Included among the top 10 most significant GO terms (FDR ≤ 1 %) are those with clear relevance to the trait, such as GO:0007595 (lactation) and GO:0043627 (response to estrogen) whereas for others the relevance is less clear, for example GO:0003785 (actin monomer binding) and GO:0010569 (regulation of double-strand break repair via homologous recombination). The complete list of enriched terms is given in Additional file 5: Table S5.

### Fat production (FAT) and fat deviation (FATD)

Significant SNPs identified for fat production (FAT) were located on BTA5 and 14 (Additional file 3: Table S1). Similarly, GWAS identified highly significant peaks for fat deviation (FATD) on BTA5, 6, 14 and 20 (Additional file 3: Table S1). Significant SNPs were mostly located on BTA14 for these two traits (Additional file 1: Figure S1 A and B).

This association analysis supports the presence of QTLs on BTA5 (76 to 107 Mb), BTA14 (0 to 20 Mb and 31 to 63 Mb), BTA20 (22 Mb and 31 to 33 Mb) related to milk production traits reported in multiple studies [9, 10, 36, 39–41]. In addition to a significant peak at 0 to 20 Mb on BTA14, the location of *DGAT1*, we identified another peak with highly significant SNPs at a genomic interval of 60 to 70 Mb associated with FATD and MILK (Additional file 3: Table S1). The presence of significant SNPs associated with milk production has been reported for this region at 63 Mb by Kolbehdari et al. [9]. Heyen et al. (1999) and Ashwell et al. [11] have also reported three regions on BTA14 at 55 and 69 Mb (between markers D14S55-ILSTS39 and D14S31-CSSM66) and 69 to 79 Mb (between markers BM4305-IRNA100), with a significant effect on fat yield and fat percentage, milk yield and protein yield respectively. The common highly significant SNPs among FAT and FATD were mostly associated with BTA14 and assigned to several known and some newly identified genes including *ARHGAP39* (BovineHD1400000188: rs134892687), *PPP1R16A* (BovineHD1400000199: rs134839376), *GRINA* (BovineHD1400000275: rs133271979), *SMPD5* (BovineHD1400000262: rs135549651) and *MROH1* (BovineHD1400000243: rs133119726).

Chromosome 5 contained the second highest number of significant SNPs for both FAT and FATD (135 and 185, respectively; genome-wise FDR ≤ 1 %). A significant peak on this chromosome was detected extending between 87 to 100 Mb in this study. This region was reported to be associated with a SNP (ss117963826) in the *GABARAPL1* gene with an antagonistic effect on milk yield and fat percentage [37]. Kolbehdari et al. [9] also reported a SNP (rs41592948) in this region linked to the *GABARAPL1* gene with an effect on dairy strength. Furthermore, we identified five significant SNPs (genome-wise FDR ≤ 5 %) within previously known QTL regions (BovineHD0500025075: rs137830740; BovineHD0500025146: rs133732696; BovineHD0500025147: rs42406616; BovineHD0500025415: rs109234621; BovineHD0500025488: rs109374096) on BTA5 for FAT trait (Additional file 4: Table S3). These SNPs are all intron variants and are located within genes *ST8SIA1*, *ABCC9*, *SLO1C1* and *PDE3A*. The QTL region that these SNPs are located in were found associated with C22:1 fatty acid content, milk fat yield and sire conception rate in Angus, Brown Swiss and Holstein cattle (Additional file 4: Table S3) [42, 43].

Several SNPs within the identified significant peak on BTA20 for FATD in this study (31–32 Mb) are located within growth hormone receptor (*GHR*) gene including BovineHD2000009236: rs109719726; ARS-BFGL-NGS-118998: rs110482506 and UA-IFASA-7069: rs41639261 (Additional file 3: Table S1). Association of significant SNPs affecting milk fat content within *GHR* gene was already reported in German Holstein-Friesian cattle [44]. This region was also reported to be associated with early embryonic survival, sire conception rate [45] and carcass weight in Holstein-Friesian cattle [33] (Additional file 4: Table S3). Another highly significant peak region for FATD was located on BTA6 and within a known QTL region (at 37–47 Mb) [46]. This region has also been reported significant for milk protein content, carcass weight and fat production in US cattle breeds [46, 47].

In the candidate SNP enrichment analysis, 1,958 SNPs were enriched in biological pathways for FAT (from 7,024 candidate SNPs P-value < 0.01). These SNPs were overrepresented in 330 significant GO terms (Additional file 5: Table S5). Included among the top 10 most significant GO terms the most relevant ones with the trait are (genome-wise FDR ≤ 1 %) GO:0051057 (positive regulation of small GTPase mediated signal transduction) and GO:0030513 (positive regulation of BMP signaling pathway). For other terms the relevance is less clear such as GO:0003281 (ventricular septum development), GO:0003279 (cardiac septum development) and GO:0003215 (cardiac right ventricle morphogenesis). The complete list of enriched GO terms for this trait is shown in Additional file 5: Table S5.

A total of 5,290 candidate SNPs (*P*-value < 0.01) associated with FATD were overrepresented in 445 enriched GO terms (Additional file 5: Table S5). Among the top seven most significant GO terms the ones with clear relevance to the FATD trait are GO:0042403 (thyroid hormone metabolic process) and GO:0035357 (peroxisome proliferator activated receptor signaling pathway), whereas the relevance is less clear for other GO terms including GO:0033158 (regulation of protein import into nucleus, translocation).

### Protein production (PROT) and protein deviation (PROTD)

Association analysis detected significant SNPs on BTA 5, 9 and 14 (Additional file 3: Table S1). The largest number of significant SNPs for protein production (PROT) was located on chromosome 14, with 41 significant SNPs (genome-wise 1 % FDR) (Additional file 1: Figure S1 C). Significant regions detected for protein deviation (PROTD) were identified on chromosomes 3, 5, 6, 10, 14, 15, 20, 26 and 29 (Additional file 3: Table S1) with the majority of significant SNPs located on chromosomes 3, 6, 14 and 20 (Additional file 1: Figure S1 D).

Our study detected strong associations on chromosome 20 from 28 to 38 Mb for PROTD. These results are consistent with previous studies reporting QTL regions surrounding *GHR* on chromosome 20 for milk yield and milk composition trait [14, 27, 41]. SNPs affecting protein yield and protein percentage on BTA20 associated with growth hormone receptor gene has been reported for several breeds including Holstein [14, 48], and Ayrshire dairy cattle [30]. We also identified highly significant SNPs including (ARS-BFGL-NGS-118998: rs110482506) in our study at 32 Mb on BTA20 which supports a QTL reported by Wang et al. [44] with a SNP downstream of the *GHR* gene.

Significant peaks were detected on chromosome 6, spanning 24 – 40 and 80 to 90 Mb for PROTD. The presence of QTLs on BTA6 in dairy cattle affecting milk production traits, near the casein gene cluster (around 87 Mb) has been reported in multiple breeds and populations including Dutch, US Holstein cattle for milk fat and milk protein yield [49–51] German Holsteins [52, 53] and Brazilian Holstein cattle [54] for milk yield and fat yield. Several highly significant SNPs (FDR ≤ 5 %) on BTA6 in this study associated with PROT and PROTD (including ARS-BFGL-NGS-42501: rs110388088; BovineHD0600006866: rs109858710; BovineHD0600009641: rs135525961; BovineHD0600009643: rs135142364; BovineHD0600009650: rs137464778) are located within a known QTL region associated with milk whey protein in dairy cattle [47]. The complete list of these SNPs is given in Additional file 4: Table S3. Also two highly significant SNPs (genome-wise FDR ≤ 1 %) (Hapmap24324-BTC-062449 and BTA-121739-no-rs) on BTA6 for PROTD in this study were reported to be very close to *ABCG2* gene (in a distance of 100 kb) [55, 56]. Variants close and within this gene were associated with protein percentage in Chinese dairy cattle [55, 56].

The peak detected for PROTD on chromosome 3 at 10 to 34 Mb is supported by previous investigations (Additional file 4: Table S3) [9, 36, 37, 57]. For example, four highly significant SNPs (genome-wise FDR ≤ 1 %) within this region (at 11 Mb) in this study (BovineHD0300003802: rs109857972; BovineHD0300003805: rs136467848; BovineHD0300003811: rs132784836 and BovineHD0300003779: rs110122034) are located within a reported QTL region associated with protein percentage in German Holstein cattle [37]. Two other SNPs in our study (BTB-00604223: rs41769311) at 52 Mb and (ARS-BFGL-NGS-4613: rs110428369) at 54 Mb on BTA15 were also found to be associated with protein percentage in Irish Holstein cattle [27].

We identified five significant SNPs in this study for PROT on BTA5 at 88 Mb including BovineHD0500025150: rs42406611; BovineHD0500025181: rs109795387 and BovineHD0500025189: rs136903701 within the gene *ABCC9*. This region was detected to be significantly associated with protein yield (at 75 to 110 Mb) in Cochran et al. [39] study.

Enrichment analysis of the candidate SNPs for PROT found 2,280 SNPs overrepresented in 658 GO terms (FDR ≤ 1 %). Included among the top 10 most highly significant GO terms (FDR ≤ 1 %) are those GO terms with a more clear relevance to the trait such as GO:0040037 (negative regulation of fibroblast growth factor receptor signaling pathway) and GO:0021903 (rostrocaudal neural tube patterning), whereas for others the relevance is less clear such as GO:0097228 (sperm principal piece). The complete list of enriched GO terms is provided in Additional file 5: Table S5.

Candidate SNP enrichment analysis for PROTD identified 447 significant GO terms potentially involved in pathways affecting protein production deviation (Additional file 5: Table S5). These enriched GO terms were overrepresented by a total number of 2,557 highly significant SNPs. The most relevance GO terms among the top 10 most significant GO terms (FDR ≤ 1 %) are GO:0007595 (lactation), GO:1902742 (apoptotic process involved in development), GO:0010257 (NADH dehydrogenase complex assembly) and GO:0033108 (mitochondrial respiratory chain complex assembly). The complete list of the significant GO terms is given in Additional file 5: Table S5.

### Fertility traits

In balanced genomic selection programs, one aim is to select bulls which sire daughters with higher reproductive performance (daughters showing an early heat in the mating period with a high probability of conception) [58]. To examine fertility, we first focused on daughter performance traits including daughter fertility and then examined the regions associated with success of insemination traits such as calving interval and days open. Association analysis did not detect any significant SNP for daughter fertility or heifer first service to calving interval (Additional file 2: Figure S2 A and B). The identified chromosomes and significant regions for CTFS and DO are discussed in detail below. In order to detect the regions with potential significant pleiotropic effects on both production and fertility traits, we also investigated the overlapping regions between these two traits (Additional files 6: Table S7 and Additional file 7: Table S8). No overlapping region was detected between production and fertility traits in this study.

### Calving to first service interval (CTFS)

A total of 20 SNPs (genome-wise FDR ≤ 5 %) were found to be associated with calving to first service interval (Fig. 1b). These SNPs were mostly located on BTA13 and 21 (Additional file 8: Table S2). The most significant SNP (genome-wise 1 % FDR) was BovineHD2100017054: rs136777407 (on BTA21), which is an intronic variant within the gene *FAM181A*.

Little is known about the function of this gene, although it was shown that methylation of this gene increases during mid-secretory phase of progesterone (P4) hormone in human endometrium [59]; This region (at 47–59 Mb) on BTA21 has not been previously reported to be associated with fertility traits in cattle or other species. The region also includes the *ASB2* gene. Therefore, these two genes can be considered as potential candidate genes affecting CTFS and calving interval associated traits in dairy cattle. The two other highly significant SNPs (genome-wise FDR ≤ 5 %) in this study on BTA21 at the same region (47–57 Mb) were BovineHD2100016620: rs136994701, within the *SLC24A4* gene, and BovineHD2100013476: rs136407309 a 3-prime UTR variant within the *NKX2-1* gene. The *SLC24A4* gene encodes a member of potassium-dependent sodium or calcium exchanger protein family and has been identified to be associated with hair color, skin pigmentation [60] and eye color in humans [61]. The gene *NKX2-1* encodes a transcription factor that regulates the expression of thyroid-specific genes (Gene ID: 7080) as well as genes that are involved in morphogenesis (Gene ID: 7080). However, no association of these two genes with fertility trait has been previously reported.

We also detected two significant SNPs associated with CTFS at 30–32 Mb on BTA13; this region has previously been reported by Sahana et al. (2010) as associated with interval from calving to first insemination and fertility index traits [17]. These two SNPs on BTA13 (BovineHD1300008936: rs136296491 and BovineHD1300009502: rs109815929) are located within candidate genes *FAM188A* and *MRC1,* respectively. The location of these SNPs is within known QTL regions associated with Inhibin level, shear force and lean meat yield in several cattle breeds (Additional file 9: Table S4). The single significant SNP on BTA5 (BovineHD0500025143: rs42718239) is located within gene *ABCC9* at 88 Mb. This region previously has been validated for calving to first insemination (ICF) trait in Nordic Holstein, Nordic Red and Jersey cattle breed but the gene *ABCC9* was not assigned to any of the associated SNPs [24]. This SNP is also located within a known QTL region associated with sire conception rate in Holstein dairy cattle [62]. The protein coded by *ABCC9* gene is thought to form ATP-sensitive potassium channels in cardiac, skeletal, vascular and non-vascular smooth muscle (Gene ID: 10060). ATP-sensitive potassium channels are expressed in many tissues and regulate different cellular functions by coupling cell metabolism with membrane potential [63]. The opening of these channels results in a flow of K^+^ ions and thus, reducing cellular excitability and contractility [64]. The K^+^ channel opening components are reported as potent inhibitors of human myometrial contractility [63]. It has been indicated that down-regulation of ATP-sensitive potassium channel (K_ATP_ channel) subunits may facilitate myometrial function during late pregnancy in humans [63]. In another study in humans, it has been revealed that estrogen may induce the activation of K_ATP_ channels to promote cell proliferation in the myometrial [65]. Therefore, the *ABCC9* gene can be considered as a potential candidate gene in dairy cattle and may have a role in cell proliferation of myometrial cells and resuming the reproduction cycle after calving.

Of 8,834 SNPs that were introduced to SNP2GO (P-value < 0.01), 2,330 SNPs were overrepresented in biological pathways and molecular functions for CTFS. These SNPs were associated with 459 significant GO terms with FDR ≤ 1 %. The GO terms with the clearest relevance for CTFS trait among the top nine most significant GO terms (FDR ≤ 1 %) are GO:0040019 (positive regulation of embryonic development) and GO:0045663 (positive regulation of myoblast differentiation), while for others the relevance is less clear, for example GO:0045836 (positive regulation of mitotic nuclear division). The complete list of significant GO terms is given in Additional file 10: Table S6.

### Days open (DO)

Association analysis identified a total of eight highly significant SNPs (genome-wise 1 % FDR) on BTA21 associated with DO (Additional file 8: Table S2; Additional file 2: Figure S2 C). These significant SNPs span the 53–59 Mb region. The peak on chromosome 21 overlaps with the one detected for CTFS, and has not been previously reported for this trait. As noted above, this peak includes gene *FAM181A*. Schulman et al. (2008) reported a QTL associated with days open EBV on chromosomes 1, 2, 5, and 25 for Finnish Ayrshire dairy cattle. These regions were not detected in our study.

The number of candidate SNPs overrepresented in the enrichment analysis was 1,897 (P-value ≤ 0.01). These SNPs were involved in 381 significant GO terms for days open (Additional file 10: Table S6). Among the top 10 most significant GO terms, the ones with clear relevance to the trait are GO:0043268 (positive regulation of potassium ion transport), GO:0071353 (cellular response to interleukin-4). Additional GO terms, for which the relationship to the trait is less clear, include GO:0000786 (nucleosome) and GO:0005070 (SH3/SH2 adaptor activity). The complete list of significant GO terms is given in Additional file 10: Table S6.

### Overlapping regions among milk production traits

The only overlapping peak among all of the production traits (MILK, FAT, FATD, PROT and PROTD) was the region identified on BTA14 (Additional file 6: Table S7). This region spans around 1.4 to 2.9 Mb and includes 74 highly significant SNPs (genome-wise FDR ≤ 1 %).

### Overlapping regions among fertility traits

Investigating overlapping regions among fertility traits has resulted in identifying eight significant SNPs on BTA21 at 53–59 Mb between two fertility traits CTFS and DO in this study (Additional file 7: Table S8). The overlapping region among these fertility traits was not reported to be associated with days open or calving to first interval traits in previous GWAS.

## Conclusion

Genome wide association analysis in this study detected several regions associated with milk production and female fertility in Canadian Holstein cattle. Most of the regions in this study were identified in other independent studies. However, novel regions of association were detected. Our result shows a novel significant region on chromosome 21 (at 47–59 Mb), which overlaps among CTFS and DO, which was not reported for fertility traits in previous association studies. This region includes several genes including *FAM181A*, *SLC24A4* and *NKX2-1*. The inclusion of several traits in one study allowed us to more easily compare overlaps that might, for example, highlight regions with pleiotropic effects. Although overlaps were observed within production and fertility traits, we did not see any overlap between production and fertility in this study. GO term enrichment analysis of the GWAS results identified terms consistent with the known physiology of the traits as well as novel or unexpected terms. The chromosomal regions identified in this study confirm several previous findings affecting production traits. Our result could also highlight new regions and pathways that may contribute to variation in fertility trait in dairy cattle. These novel regions can be used for further functional analysis to identify genes, gene networks and variants that explain variation in these traits.

## Methods

### Animals and data

A population of 3,729 North American Holstein bulls was used in this study, which examined nine production and fertility traits: milk production (MILK), fat production (FAT), protein production (PROT), fat and protein deviation (FATD and PROTD respectively), daughter fertility (DF), first service to calving interval (FSTCh), calving to first service interval (CTFS), and days open (DO). The Canadian Dairy Network (CDN) provided available pedigree, genotypes and official evaluations for proven bulls born between 1956 and 2009. Individuals were genotyped using the BovineSNP50K (50 k) panel (3,729 bulls) (Illumina, San Diego, CA) or the high density (HD, 777 k) SNP panel (2,387 bulls), respectively. In this work only autosomal SNPs were included. For 50 k panel, SNP list used for official genomic evaluation by CDN was considered. These SNPs have passed standard quality control measures used by CDN. Quality control (QC) was performed on the HD genotyping data using snp1101 software (Mehdi Sargolzaei, personal communication) and 116,619 SNPs were excluded on the basis of Mendelian error rate higher > 0.05 (3,566 SNPs), low call rate < 0.9 (6,446 SNPs), with low MAF (1e-06 – 0.5; 61,577 SNPs), excess of heterozygosity > 0.15 (90 SNPs) and excluded by user (46,433 SNPs excluded from sex chromosome and misplaced SNPs). The number of SNPs kept in the analyses before imputation was 40,666 and 657,986 SNPs for 50 k and HD panels, respectively.

Genotypes of 3,729 50 k animals were imputed to HD with a reference population of 2,387 HD individuals using FImpute V2.2 software [66]. Quality control (QC) was performed on the imputed data as well. SNPs with minor allele frequency (MAF) of less than 1 % (55,817 SNPs) and high Mendelian error rate more than 5 % (74 SNPs) were excluded. After quality control, 602,095 SNPs remained for use in the subsequent association analysis.

### Calculating de-regressed proofs

In this study de-regressed Holstein bull proofs were used as pseudo phenotypes. A bull’s published estimated breeding value (EBV) is a weighted mean of his daughters deviations (DD) and his parental average (PA) [67]. The de-regressed bull proofs were computed by CDN as shown below [67]:

$$ D{E}_{prg}=\frac{Re{l}_{EBV}}{1-Re{l}_{EBV}}-\frac{Re{l}_{PA}}{1-Re{l}_{PA}} $$

$$ Re{l}_{DD}=\frac{D{E}_{prg}}{D{E}_{prg}+1} $$

$$ DEBV=PA+\frac{\left(EBV\mathit{\hbox{-}}PA\right)}{Re{l}_{DD}} $$

Where, *DE*_*prg*_ is the daughter equivalent from progeny information, *Rel*_*EBV*_ and *Rel*_*PA*_ are the reliabilities of EBV and PA, respectively, *Rel*_*DD*_ is the reliability of DD, and DEBV is the de-regressed bull proof.

### Genome wide association analysis (GWAS)

Association analysis was performed using a single SNP regression mixed linear model implemented in the snp1101 software [68]. The mixed linear model was:

$$ {Y}_i=\mu +\beta {g}_i+{a}_i+{e}_i $$

where *Y*_*i*_ is pseudo phenotype of the i^th^ bull (de-regressed bull proofs, DEBV); *μ* is the overall mean; *β* is the linear regression coefficient (allele substitution effect) of the SNP; *g*_*i*_ is the SNP genotype of the i^th^ bull, which was coded as 0, 1 and 2 for SNP genotypes BB, AB and AA, respectively; *α*_*i*_ is the random additive polygenic effect of the i^th^ bull and *e*_i_ is the random error term.

Assumptions for the model are *a*_*i*_ : *a* ~ *N*(0, *Gσ*_*a*_^2^) where G is the genomic relationship matrix [68] and *σ*_*a*_^2^ is the polygenic additive genetic variance; *e*_*i*_ : *e* ~ *N*(0, *Rσ*_*e*_^2^) where *σ*_*e*_^2^ is the residual variance. R is a diagonal matrix containing weights for the residual variance based on the reliabilities of the de-regressed bull proofs [68].

To account for multiple tests, 5 % and 1 % genome-wise false discovery rate (FDR) were used to identify significant and highly significant associations, respectively. The inflation factor λ [69] and quantile-quantile (Q-Q) plots were calculated to compare observed distributions of –log (*P*-value) to the expected distribution under the no association model for each trait.

### Candidate SNP enrichment analysis

The SNP2GO R package was used for functional analysis of the genome-wide association results [25]. Genomic annotations, associated Gene Ontology terms (GO terms) and the list of significant (*P*-value < 0.01) and non-significant SNPs (termed “candidate” and “non-candidate” SNPs in the SNP2GO documentation) for each trait were provided as input to SNP2GO, which reports biological pathways or processes that are enriched for significant SNPs. For this analysis, the Ensembl version 78 genomic annotation file for *Bos taurus* UMD 3.1 assembly was used in conjunction with the Ensembl gene ID file from Ensembl version 78, which contains gene ID to GO term associations. The SNP2GO “extension” value was set to 50 nucleotides, which expands the gene region (by 50 nucleotides upstream and 50 nucleotides downstream) when identifying overlaps between genes and markers. The “runs” parameter was set to 100,000 and a false discovery rate (FDR) of 1 % was used to correct for multiple testing.

## Abbreviations

BTA, Bos taurus autosome; CTFS, calving to first service interval; DF, daughter fertility; DO, days open; EBV, estimated breeding value; FAT, fat production; FATD, fat deviation; FDR, false discovery rate; FSTCh, heifer first service to calving interval;GO, gene ontology; GS, genomic selection; GWAS, genome-wide association study; LD, linkage disequilibrium; MILK, milk production; PROT, protein production; PROTD, protein deviation; QC, quality control; QTL, quantitative trait loci; SNP, single nucleotide polymorphism

## Additional files

Additional file 1: Figure S1. Genome-wide association analysis and quantile-quantile (Q-Q) of P-values of SNPs from single SNP regression mixed linear model for milk production traits: Panels A-D: The –log_10_ of the P-value for association with SNPs is plotted. Chromosome number is shown on the horizontal axis. The traits included in this file are A. fat production (FAT); B. fat deviation (FATD); C. protein production (PROT); D. protein deviation (PROTD). The red line is the threshold for significant SNPs at 1 % FDR. The green line is the threshold for significant SNPs at 5 % FDR. Panels E-I: In the Q-Q plots the blue dots represent the –log_10_(P-values) to the expected distribution under the null hypothesis of no association. The traits are shown as E. milk production (MILK); F. fat production (FAT); G. fat deviation (FATD); H. protein production (PROT); I. protein deviation (PROTD). The red line denotes the expected pattern under the null hypothesis. Deviations between the red line and blue dots indicate how the test statistics of loci deviate from the null hypothesis. (PPTX 971 kb) Additional file 2: Figure S2. Genome-wide association analysis and quantile-quantile (Q-Q) of P-values of SNPs from single SNP regression mixed linear model for fertility traits: Panels A-C: The –log_10_ of the P-value for association with SNPs is plotted. Chromosome number is shown on the horizontal axis. The traits are shown as A. daughter fertility (DF); B. heifer first service to calving interval (FSTCh); C. days open (DO). The red line is the threshold for significant SNPs at 1 % FDR. The green line is the threshold for significant SNPs at 5 % FDR. Panels D-G: In the Q-Q plots the blue dots represent the –log_10_(P-values) to the expected distribution under the null hypothesis of no association. The traits are shown as D. daughter fertility (DF); E. heifer first service to calving interval (FSTCh); F. calving to first service interval (CTFS); G. days open (DO). The red line denotes the expected pattern under the null hypothesis. Deviations between the red line and blue dots indicate how the test statistics of loci deviate from the null hypothesis. (PPTX 3218 kb) Additional file 3: Table S1. Excel file describing highly significant SNPs (genome-wise FDR _≤_ 5 %, FDR ≤ 1% and genome-wise Bonferroni ≤ 5 %) resulting from GWAS using single SNP regression mixed linear model for milk production (MILK), fat production (FAT), fat deviation (FATD), protein production (PROT) and protein deviation (PROTD). (XLS 518 kb) Additional file 4: Table S3. Excel file describing previously identified production or fertility trait QTL that overlap with the SNPs identified in this study for milk production (MILK), fat production (FAT), fat deviation (FATD), protein production (PROTD) and protein deviation (PROTD). (XLS 4775 kb) Additional file 5: Table S5. Excel file describing gene ontology biological process terms enriched among GWAS results for production traits; milk production (MILK), fat production (FAT), fat deviation (FATD), protein production (PROT) and protein deviation (PROTD). (XLS 803 kb) Additional file 6: Table S7. Common significant SNPs (genome-wise 1 % FDR) identified among all production related traits (MILK, FAT, PROT, FATD, PROTD) using single SNP regression mixed linear model on imputed BovineHD (777 k) genotypes in Holstein cattle. (XLS 43 kb) Additional file 7: Table S8. Common significant SNPs (genome-wise 1 % FDR) identified among all fertility related traits (CTFS and DO) using single SNP regression mixed linear model on the imputed BovineHD (777 k) genotypes in Holstein cattle. (XLS 34 kb) Additional file 8: Table S2. Excel file describing highly significant SNPs (genome-wise FDR _≤_ 5 %, FDR ≤ 1% and genome-wise Bonferroni ≤ 5 %) resulting from GWAS using single SNP regression mixed linear model for calving to first service interval (CTFS) and days open (DO). (XLS 40 kb) Additional file 9: Table S4. Excel file describing previously identified production or fertility trait QTL that overlap with the SNPs identified in this study for calving to first service interval (CTFS). (XLS 30 kb) Additional file 10: Table S6. Excel file describing gene ontology biological process terms enriched among GWAS results for fertility traits; calving to first service interval (CTFS) and days open (DO). (XLS 339 kb)
