# Supplementary material for: Genome-wide association for milk production and female fertility traits in Canadian dairy Holstein cattle
Source: BMC Genet. 2016 Jun 10;17:75. doi: 10.1186/s12863-016-0386-1 (PMC4901445; doi:10.1186/s12863-016-0386-1)
Supplement: Additional file 1: Figure S1. — Genome-wide association analysis and quantile-quantile (Q-Q) of P-values of SNPs from single SNP regression mixed linear model for milk production traits: Panels A-D: The –log10 of the P-value for association with SNPs is plotted. Chromosome number is shown on the horizontal axis. The traits included in this file are A. fat production (FAT); B. fat deviation (FATD); C. protein production (PROT); D. protein deviation (PROTD). The red line is the threshold for significant SNPs at 1 % FDR. The green line is the threshold for significant SNPs at 5 % FDR. Panels E-I: In the Q-Q plots the blue dots represent the –log10(P-values) to the expected distribution under the null hypothesis of no association. The traits are shown as E. milk production (MILK); F. fat production (FAT); G. fat deviation (FATD); H. protein production (PROT); I. protein deviation (PROTD). The red line denotes the expected pattern under the null hypothesis. Deviations between the red line and blue dots indicate how the test statistics of loci deviate from the null hypothesis. (PPTX 971 kb) [file 12863_2016_386_MOESM1_ESM.pptx]

## Slide 1
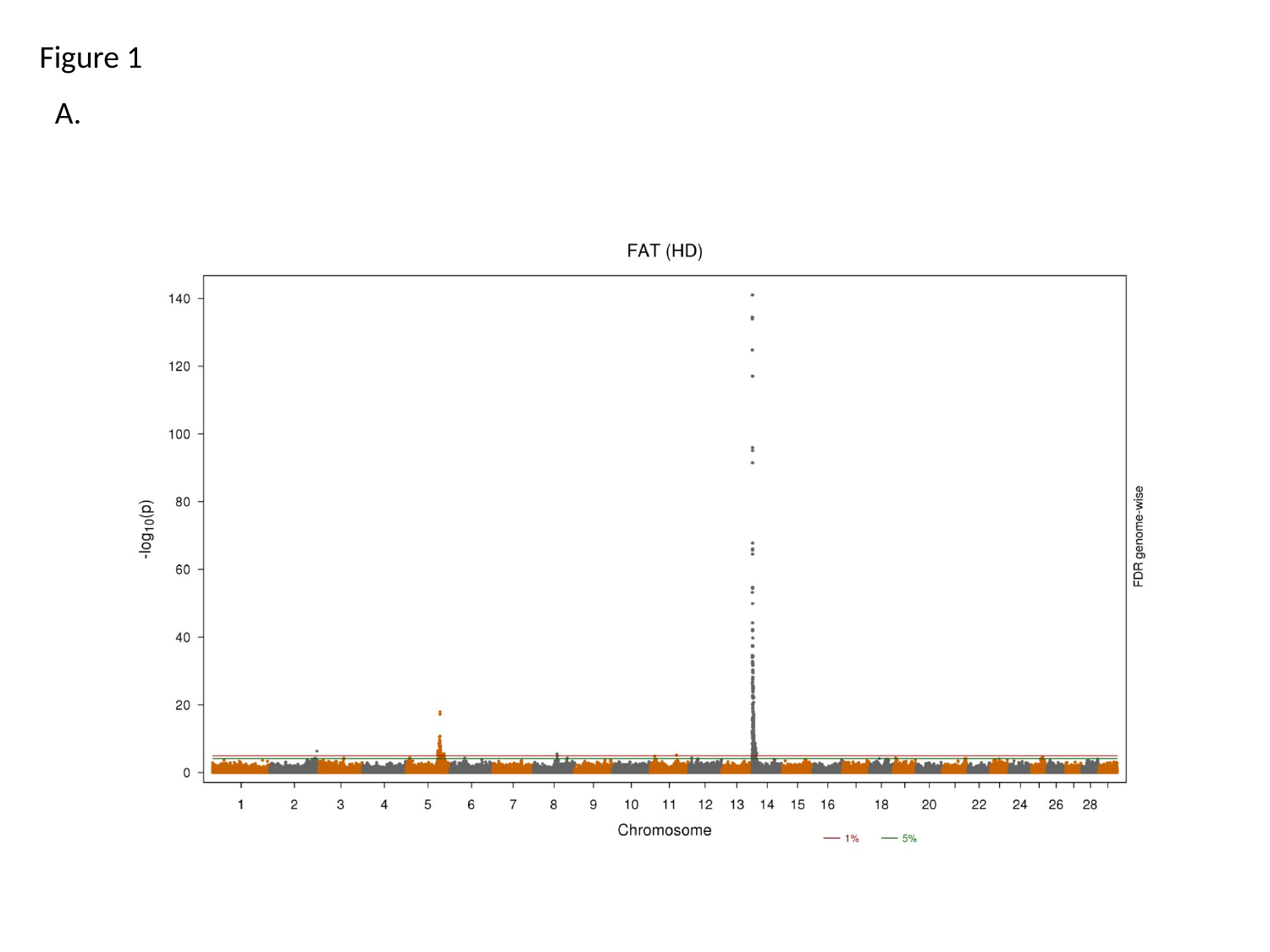

Figure 1
A.

## Slide 2
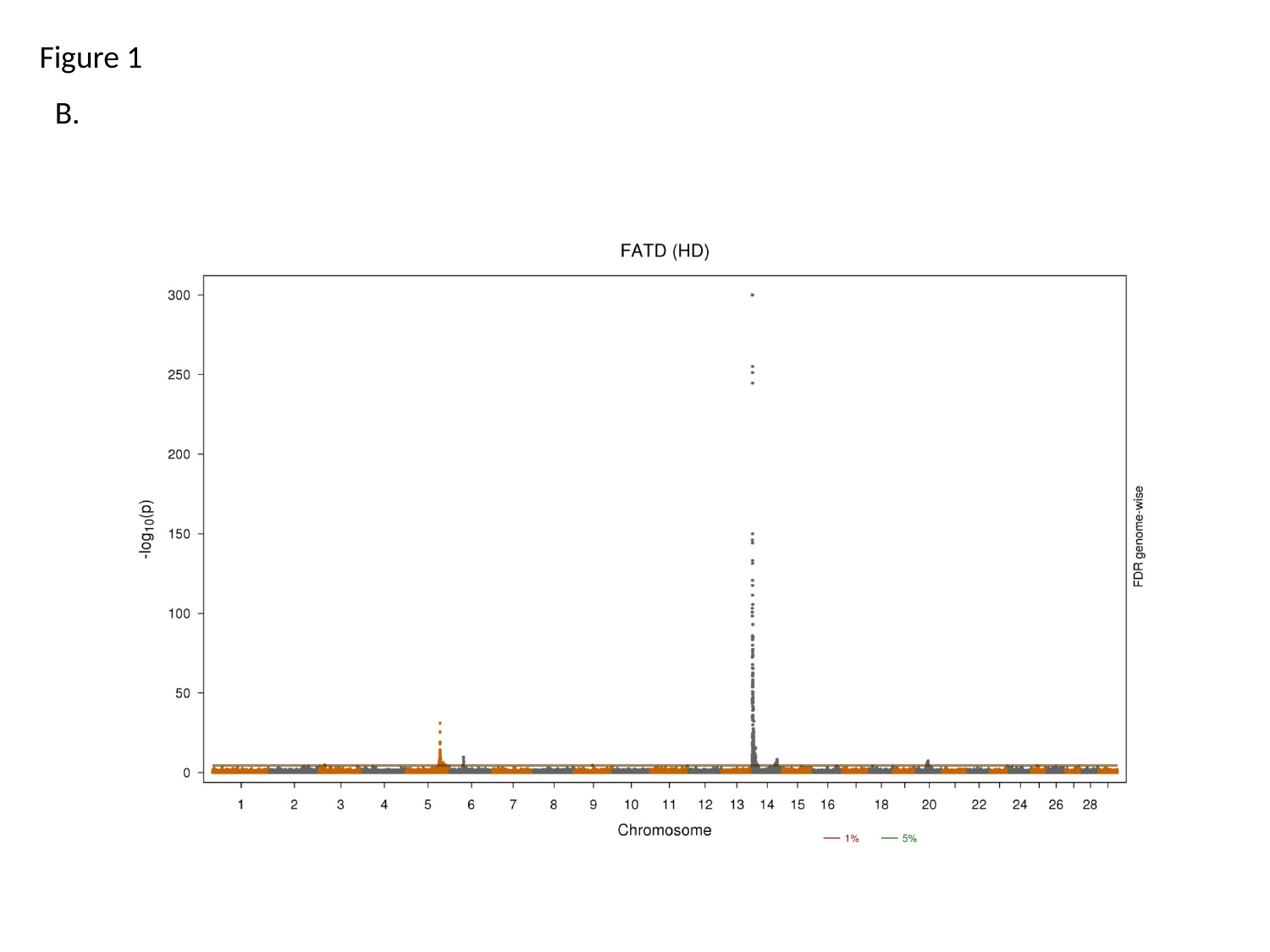

Figure 1
B.

## Slide 3
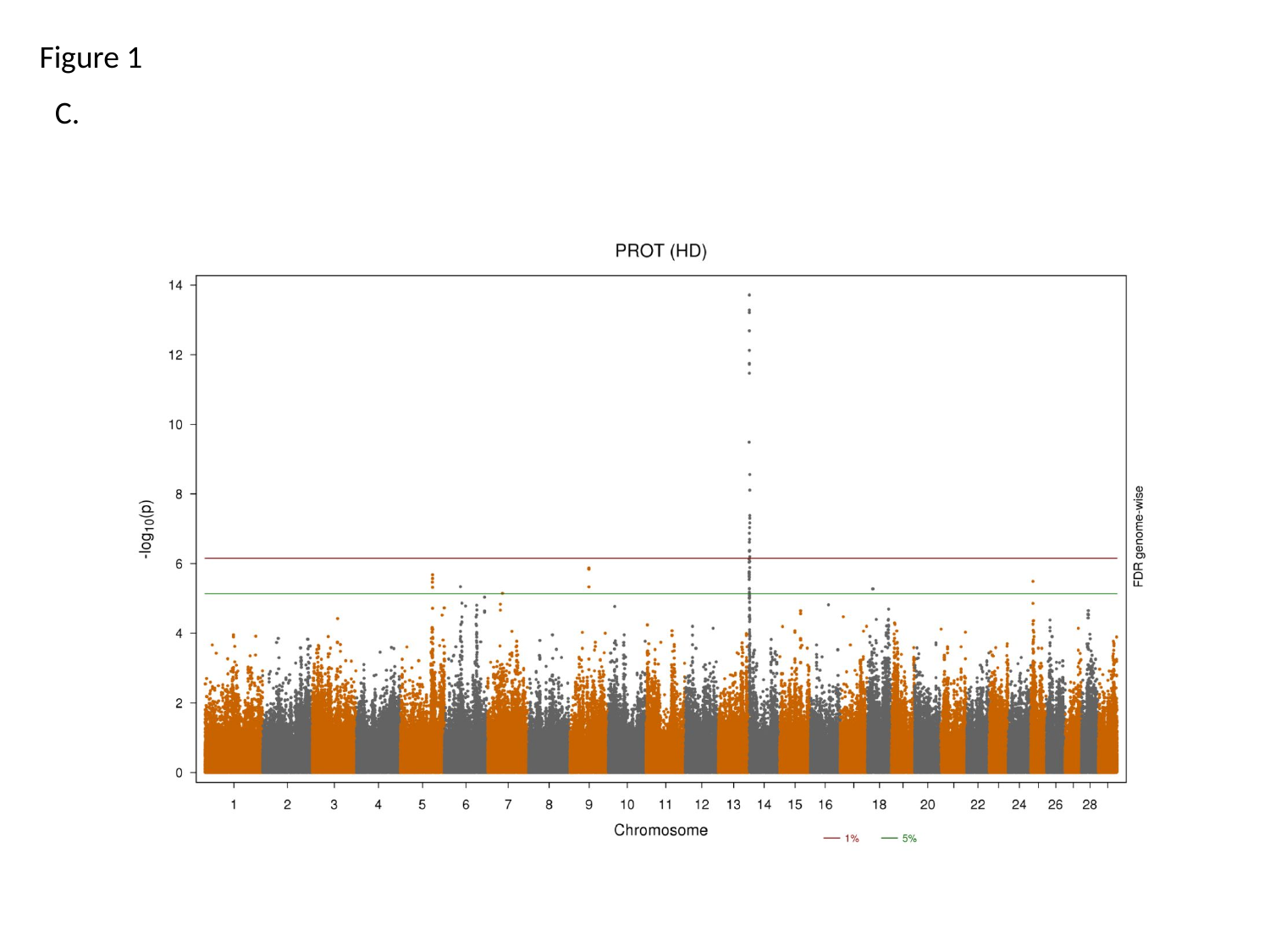

Figure 1
C.

## Slide 4
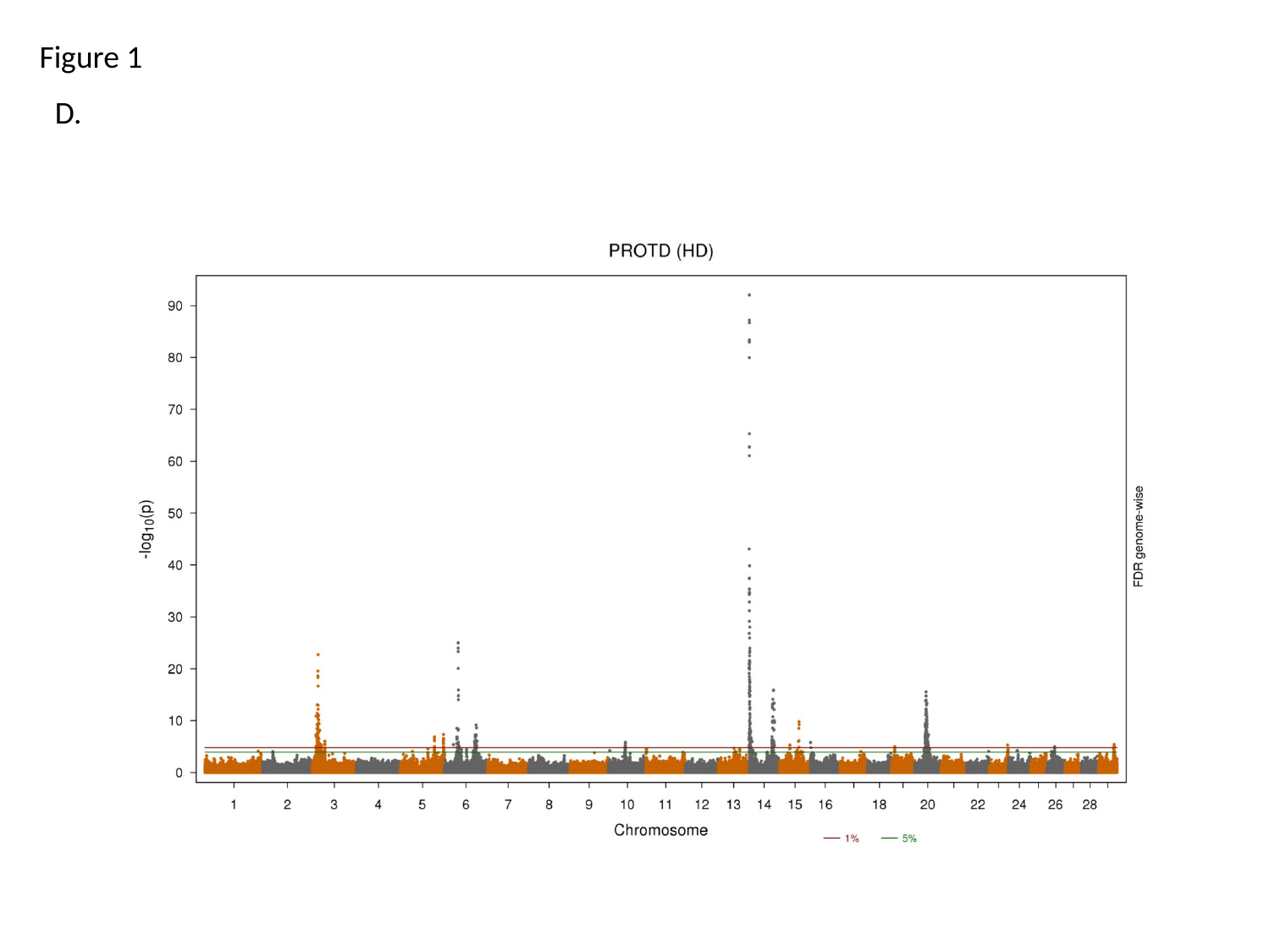

Figure 1
D.

## Slide 5
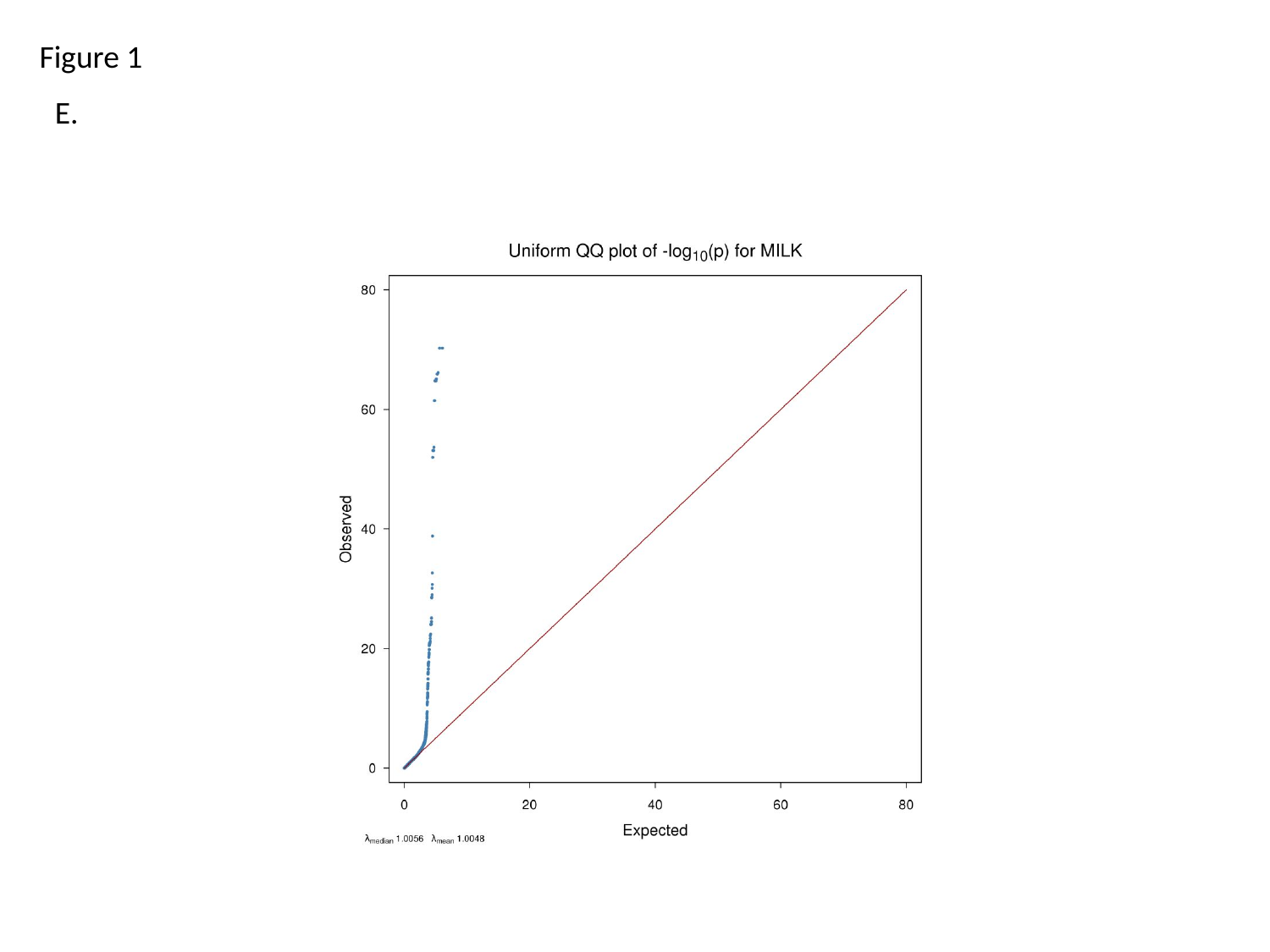

Figure 1
E.

## Slide 6
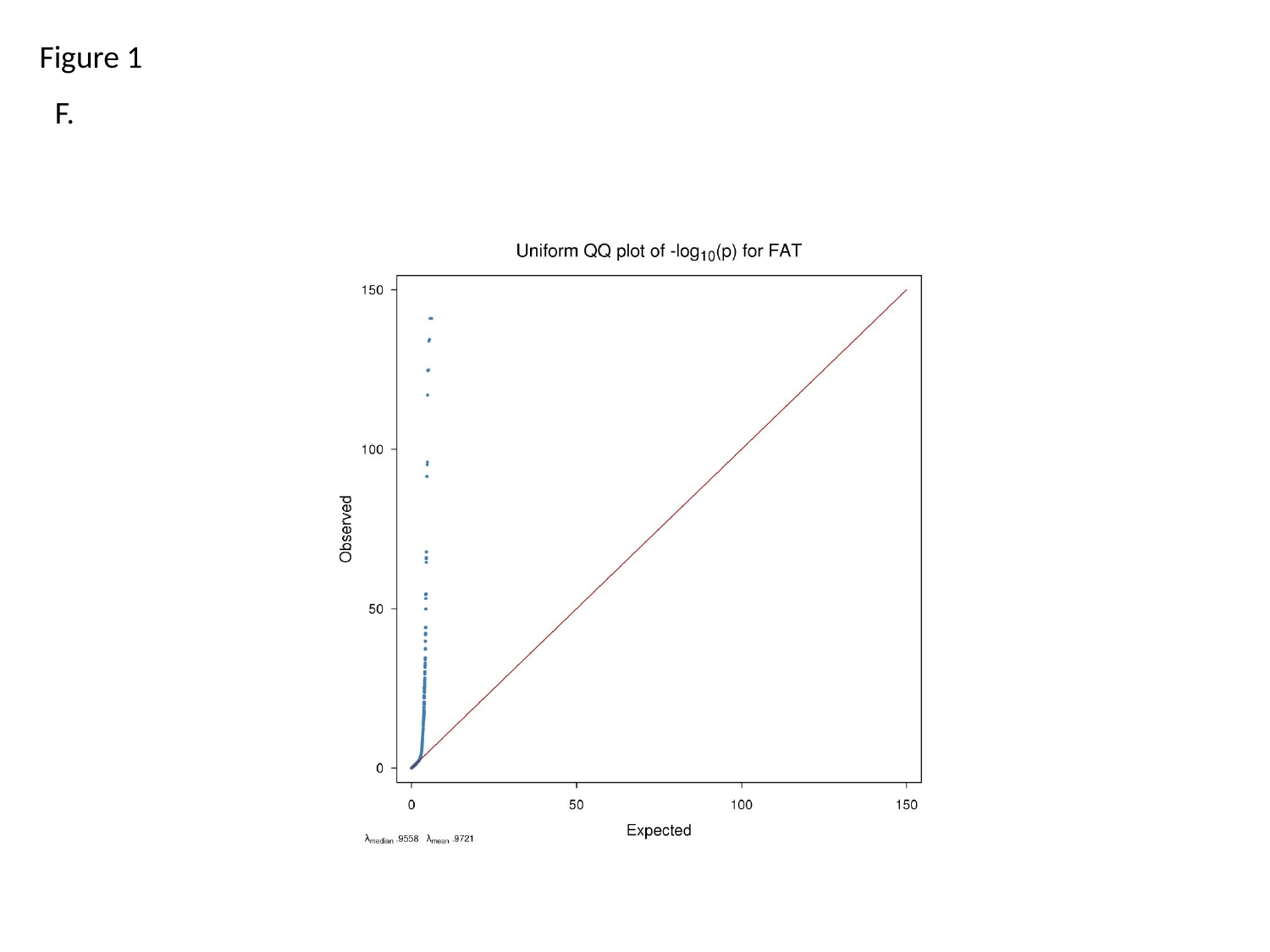

Figure 1
F.

## Slide 7
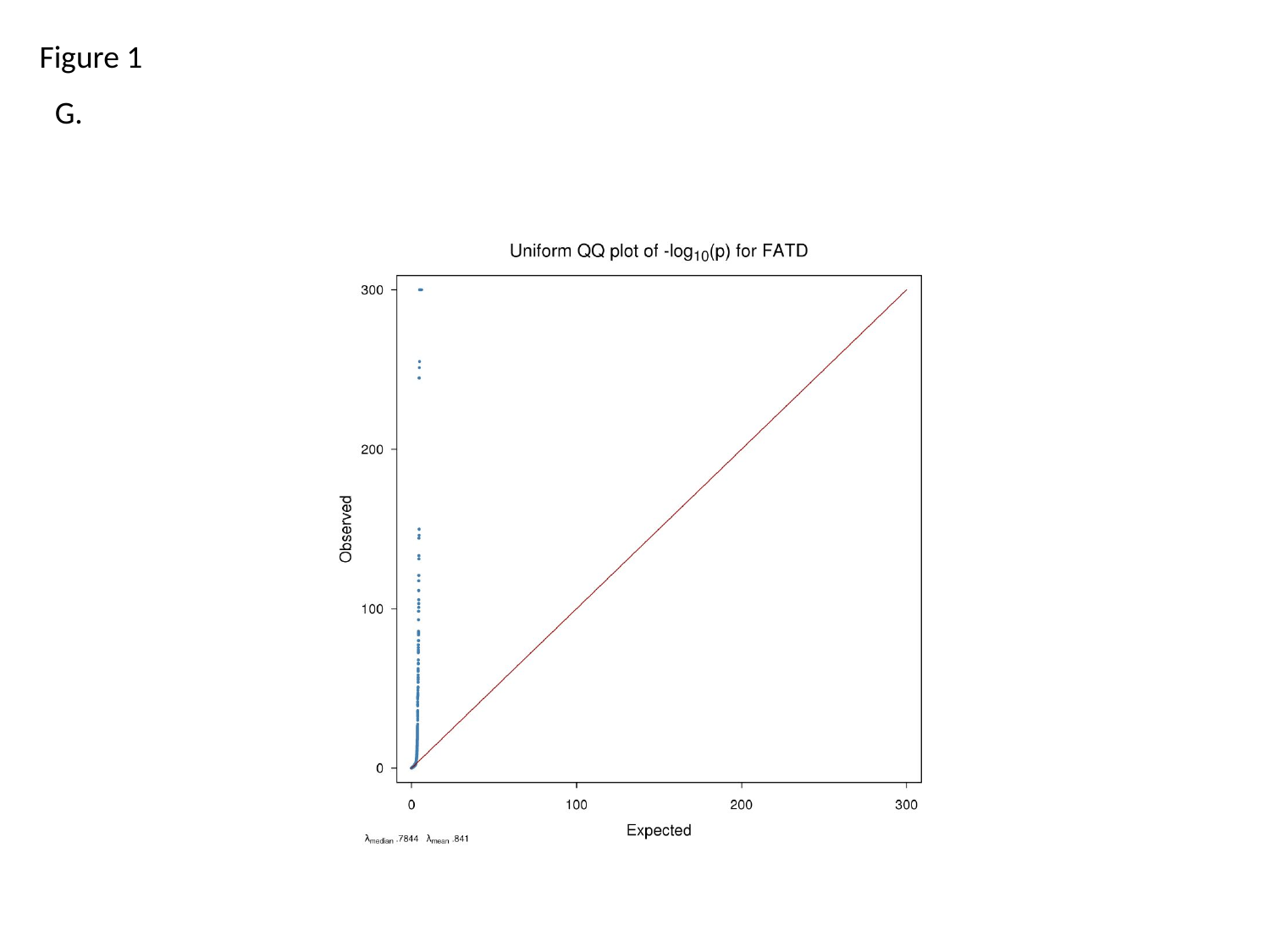

Figure 1
G.

## Slide 8
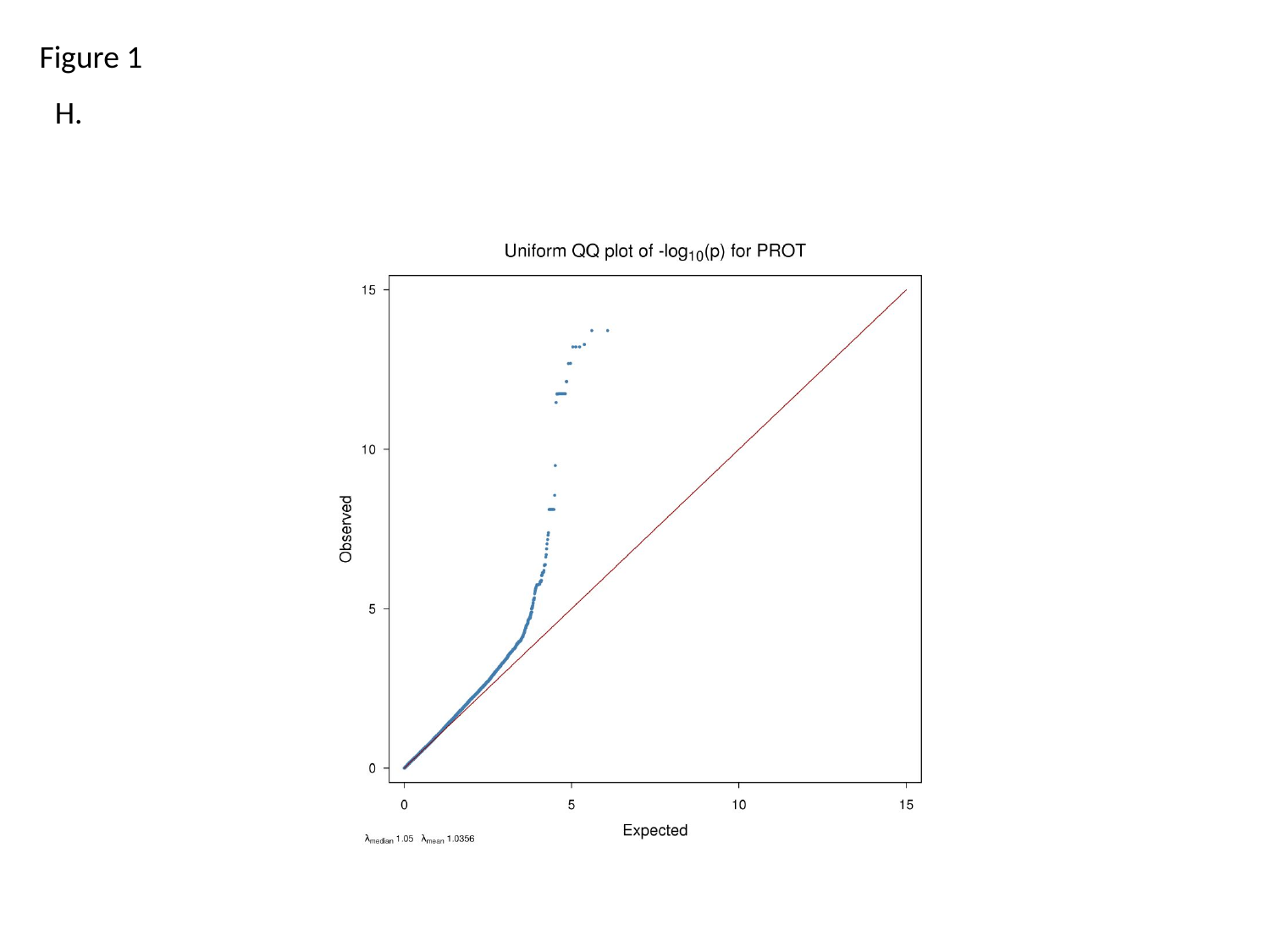

Figure 1
H.

## Slide 9
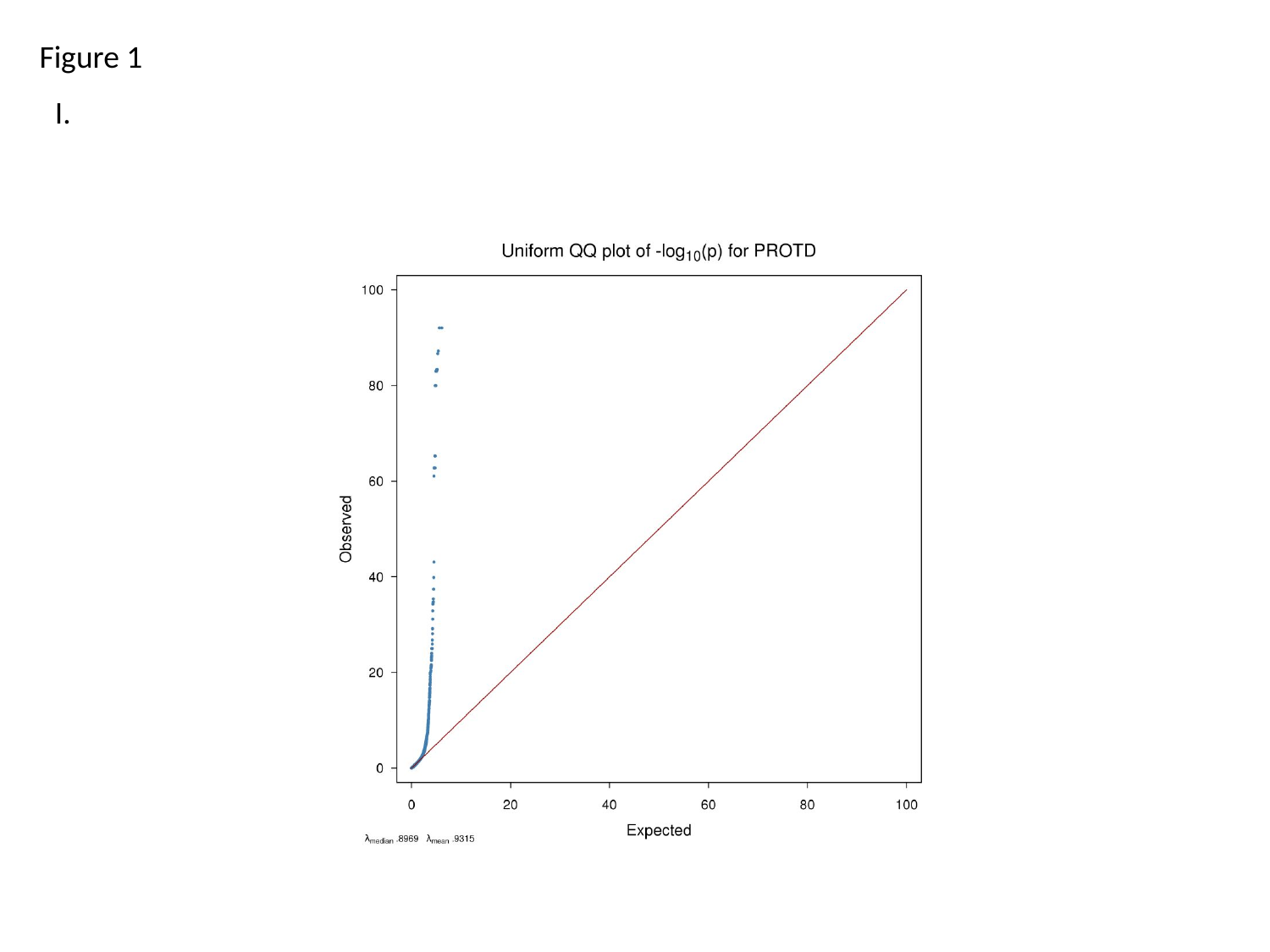

Figure 1
I.
