# Supplementary material for: Genome-wide association for milk production and female fertility traits in Canadian dairy Holstein cattle
Source: BMC Genet. 2016 Jun 10;17:75. doi: 10.1186/s12863-016-0386-1 (PMC4901445; doi:10.1186/s12863-016-0386-1)
Supplement: Additional file 2: Figure S2. — Genome-wide association analysis and quantile-quantile (Q-Q) of P-values of SNPs from single SNP regression mixed linear model for fertility traits: Panels A-C: The –log10 of the P-value for association with SNPs is plotted. Chromosome number is shown on the horizontal axis. The traits are shown as A. daughter fertility (DF); B. heifer first service to calving interval (FSTCh); C. days open (DO). The red line is the threshold for significant SNPs at 1 % FDR. The green line is the threshold for significant SNPs at 5 % FDR. Panels D-G: In the Q-Q plots the blue dots represent the –log10(P-values) to the expected distribution under the null hypothesis of no association. The traits are shown as D. daughter fertility (DF); E. heifer first service to calving interval (FSTCh); F. calving to first service interval (CTFS); G. days open (DO). The red line denotes the expected pattern under the null hypothesis. Deviations between the red line and blue dots indicate how the test statistics of loci deviate from the null hypothesis. (PPTX 3218 kb) [file 12863_2016_386_MOESM2_ESM.pptx]

## Slide 1
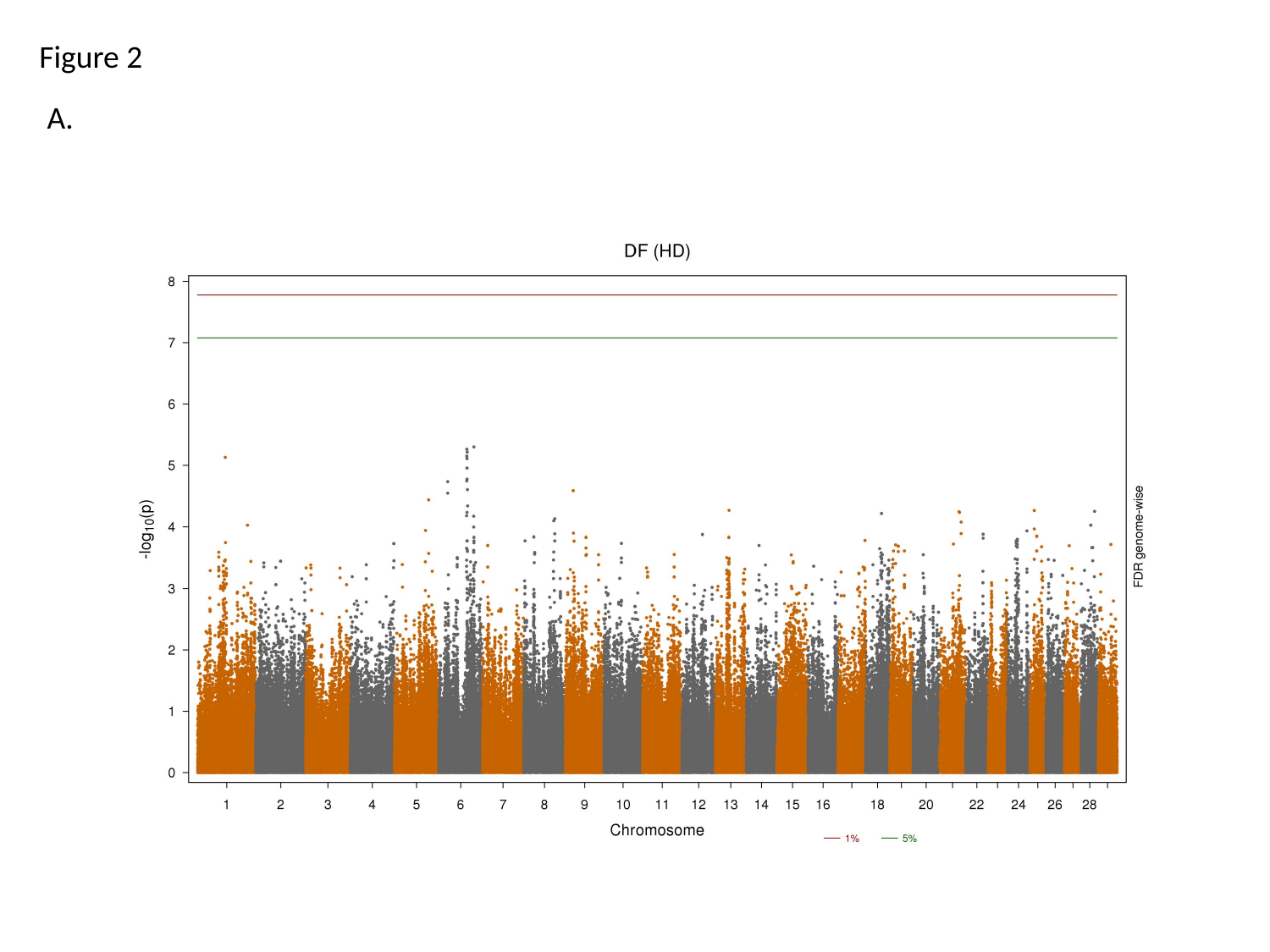

Figure 2
A.

## Slide 2
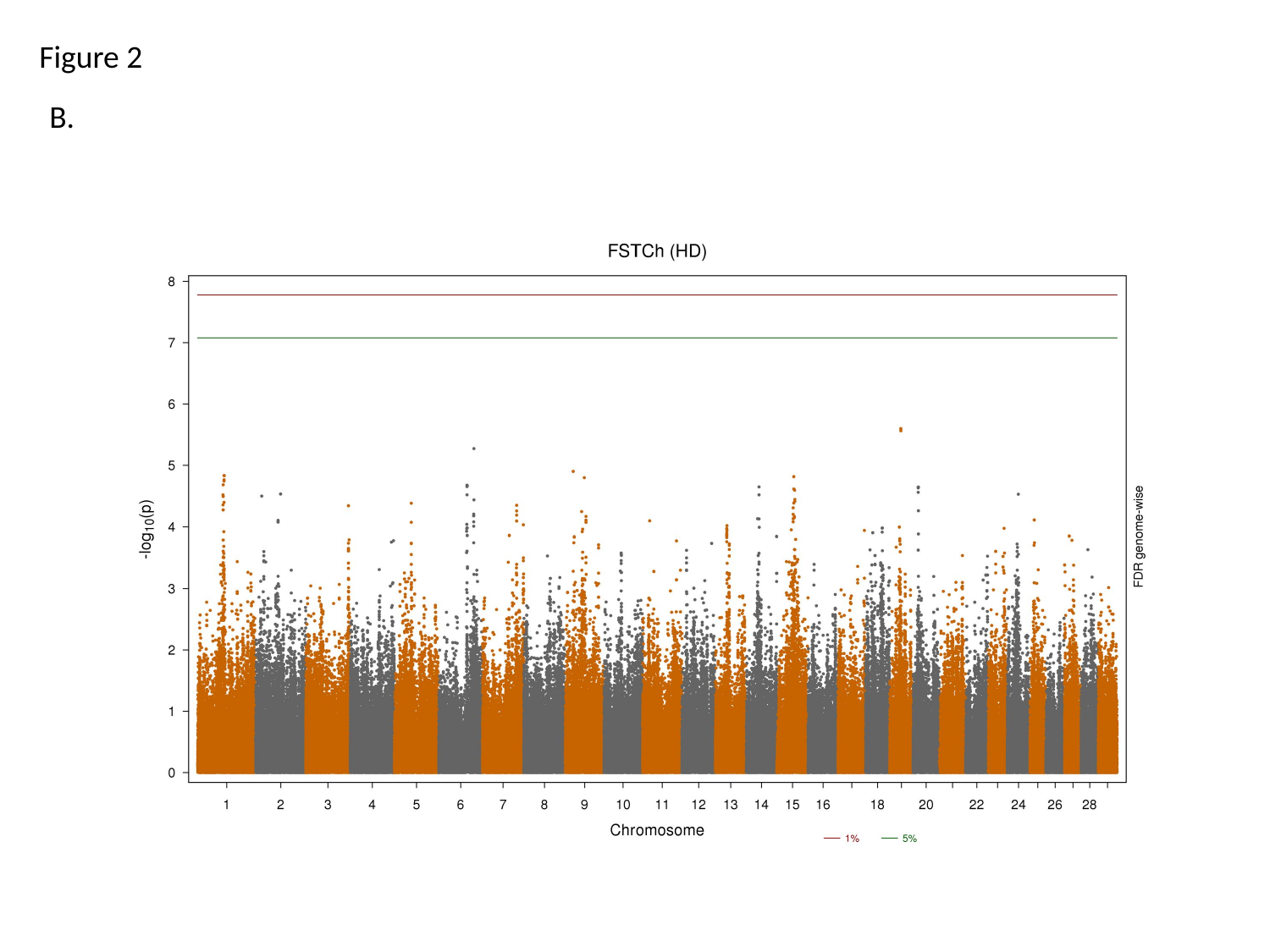

Figure 2
B.

## Slide 3
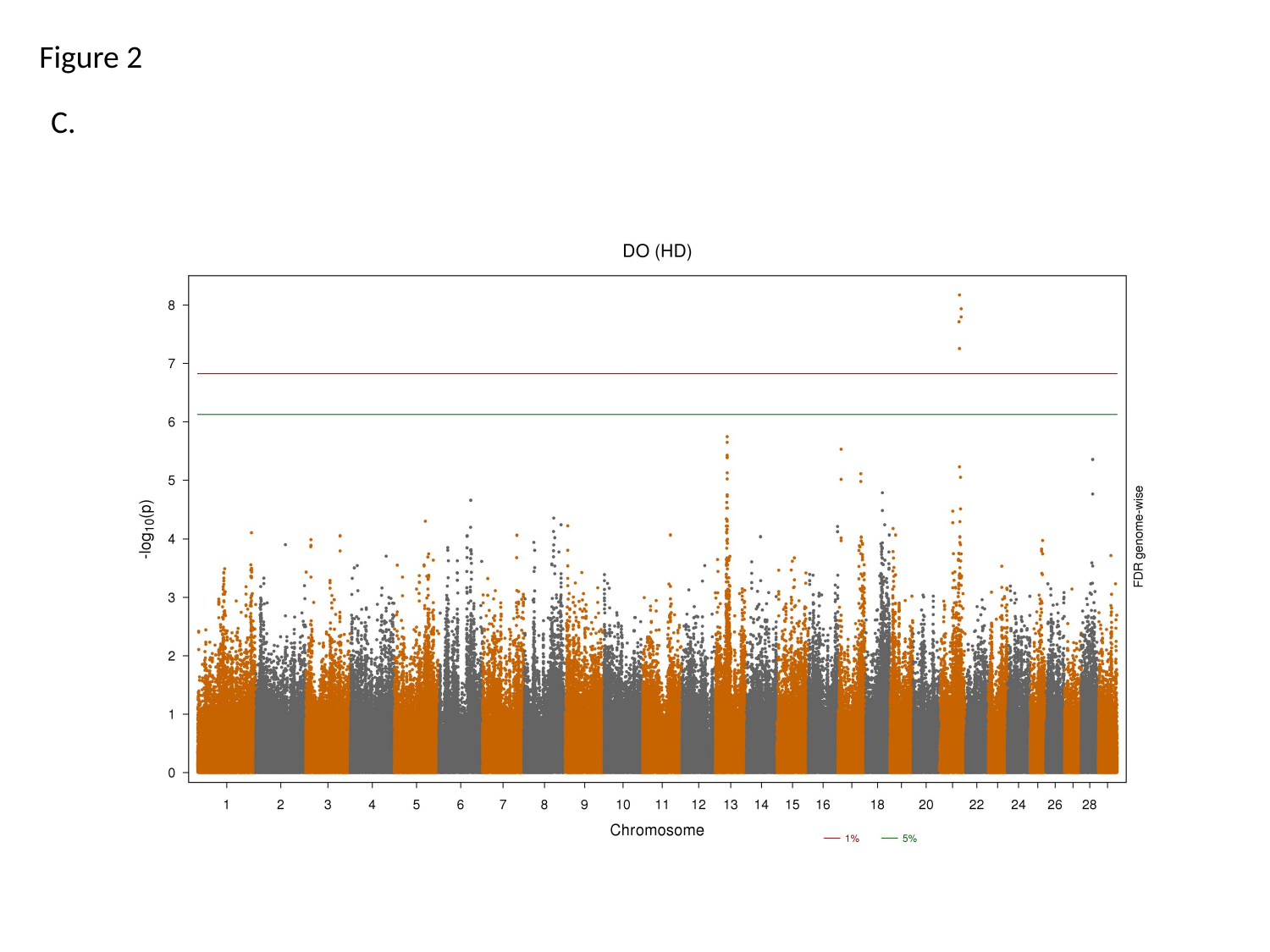

Figure 2
C.

## Slide 4
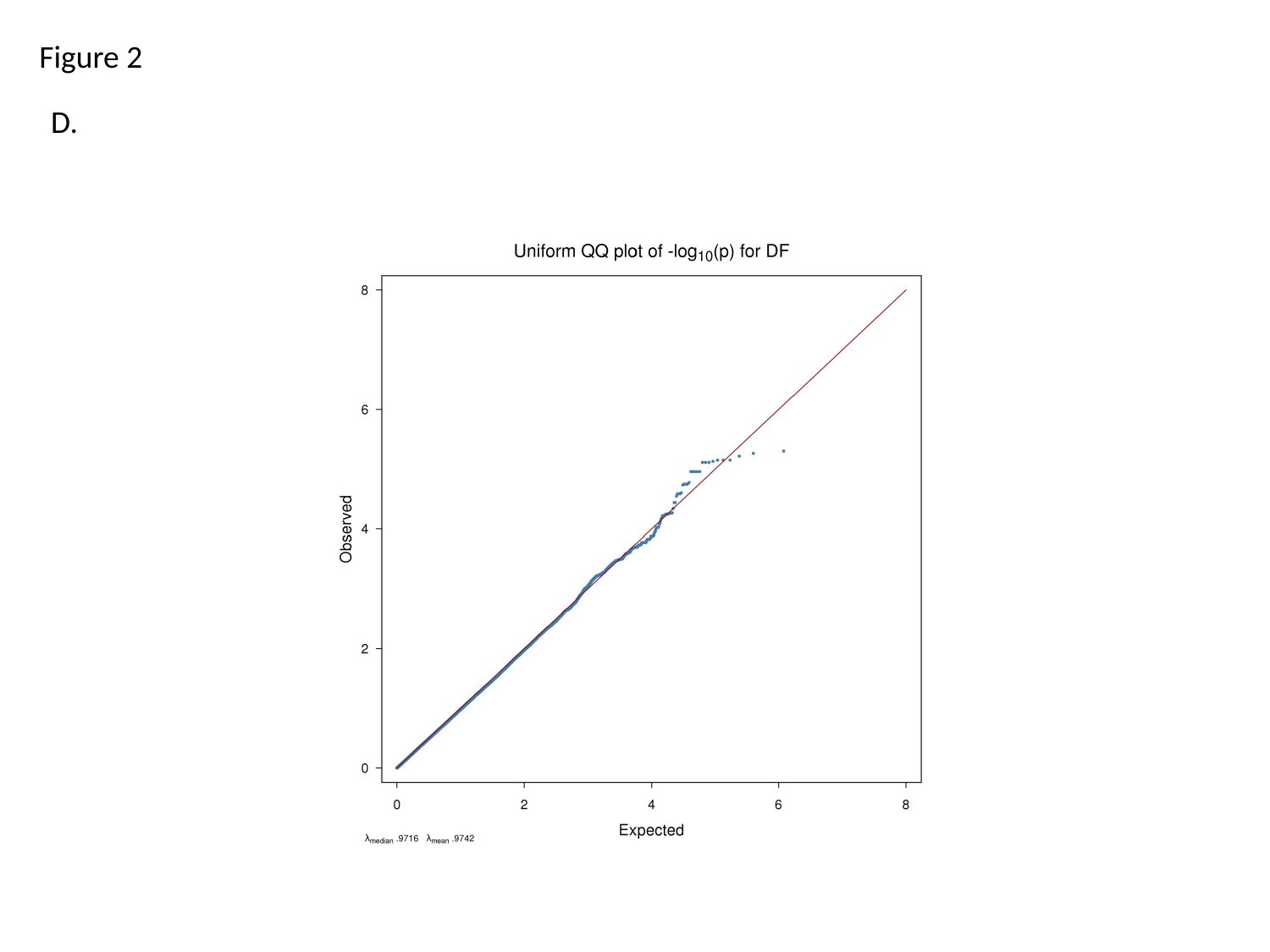

Figure 2
D.

## Slide 5
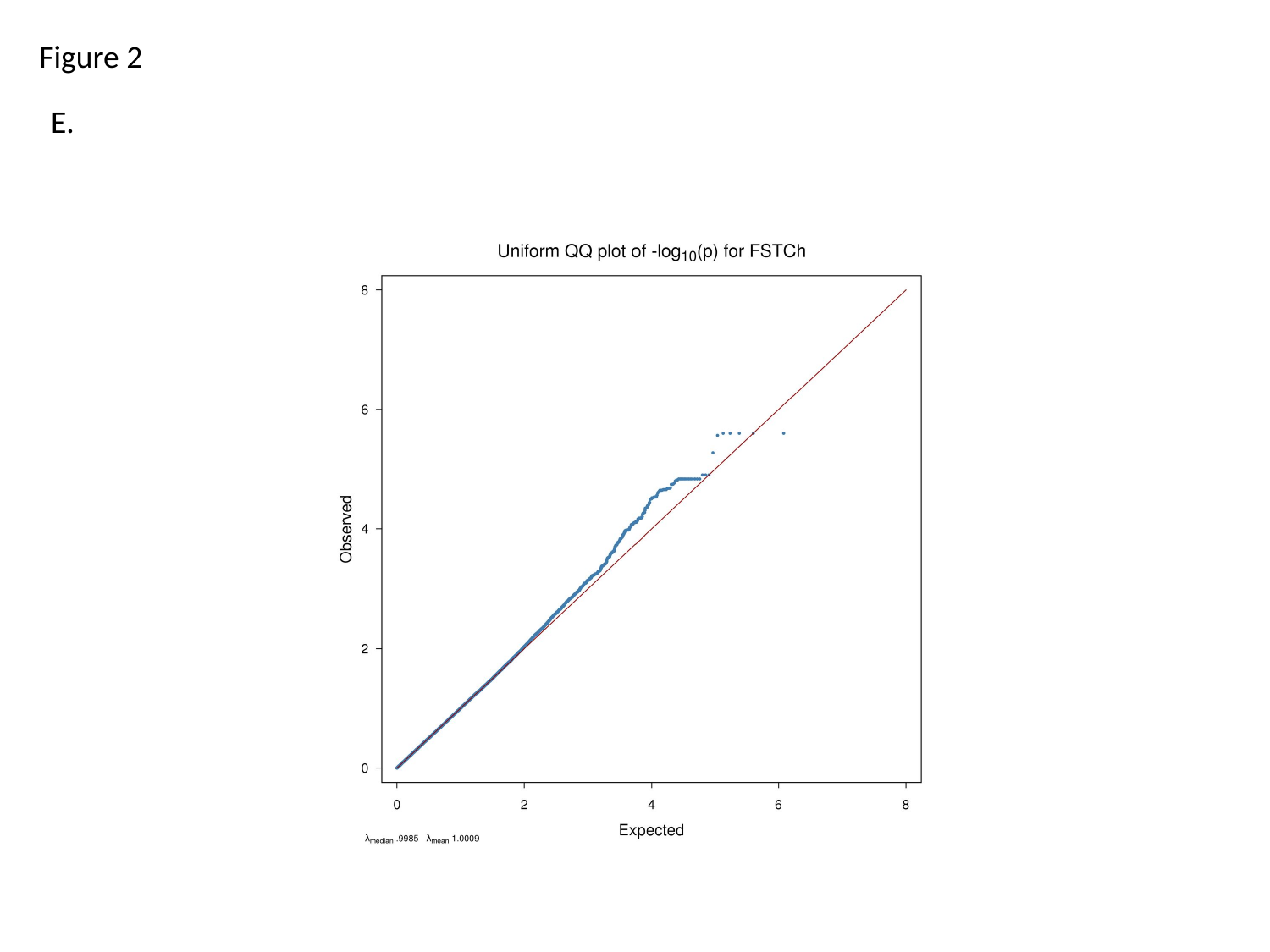

Figure 2
E.

## Slide 6
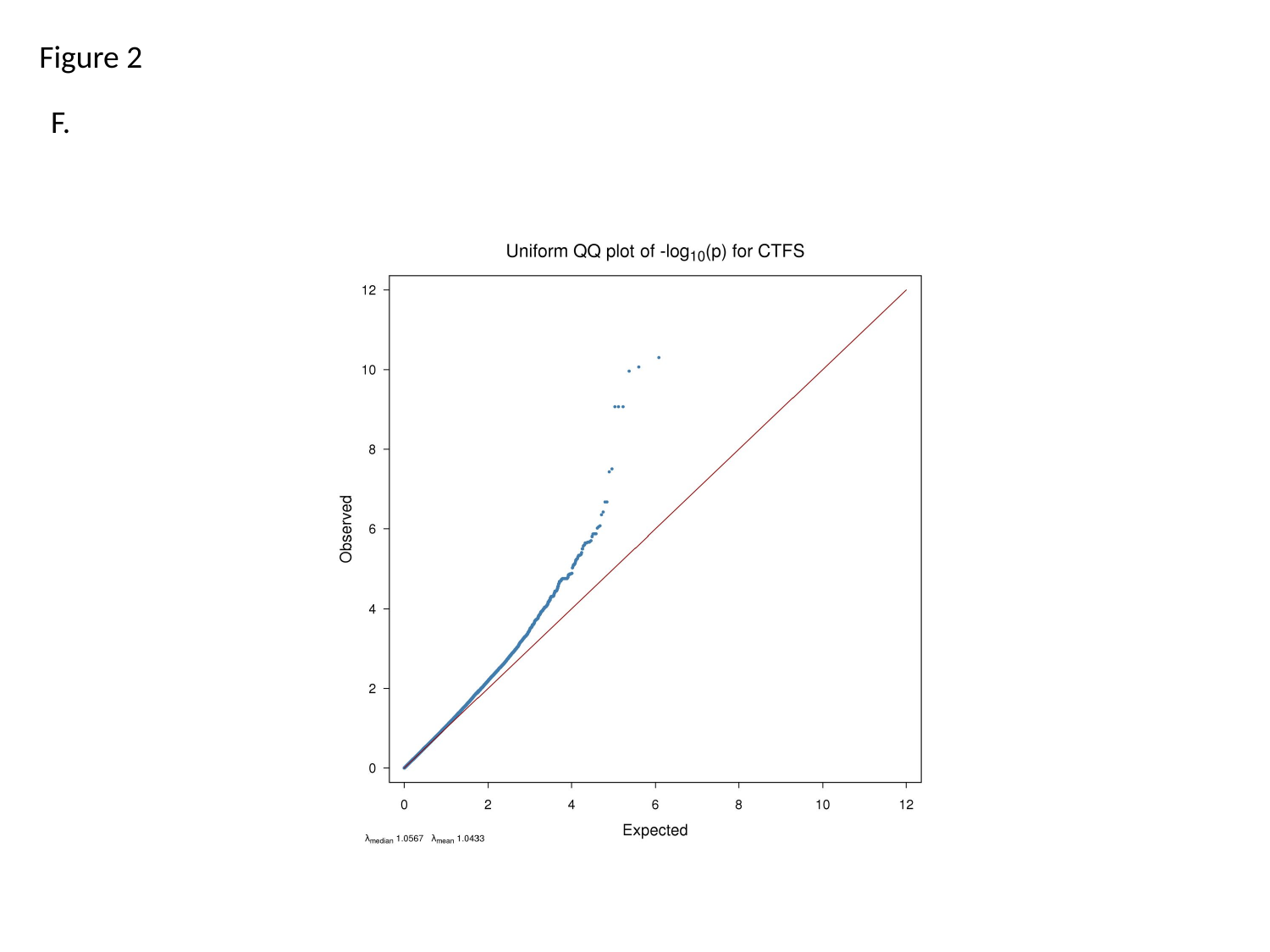

Figure 2
F.

## Slide 7
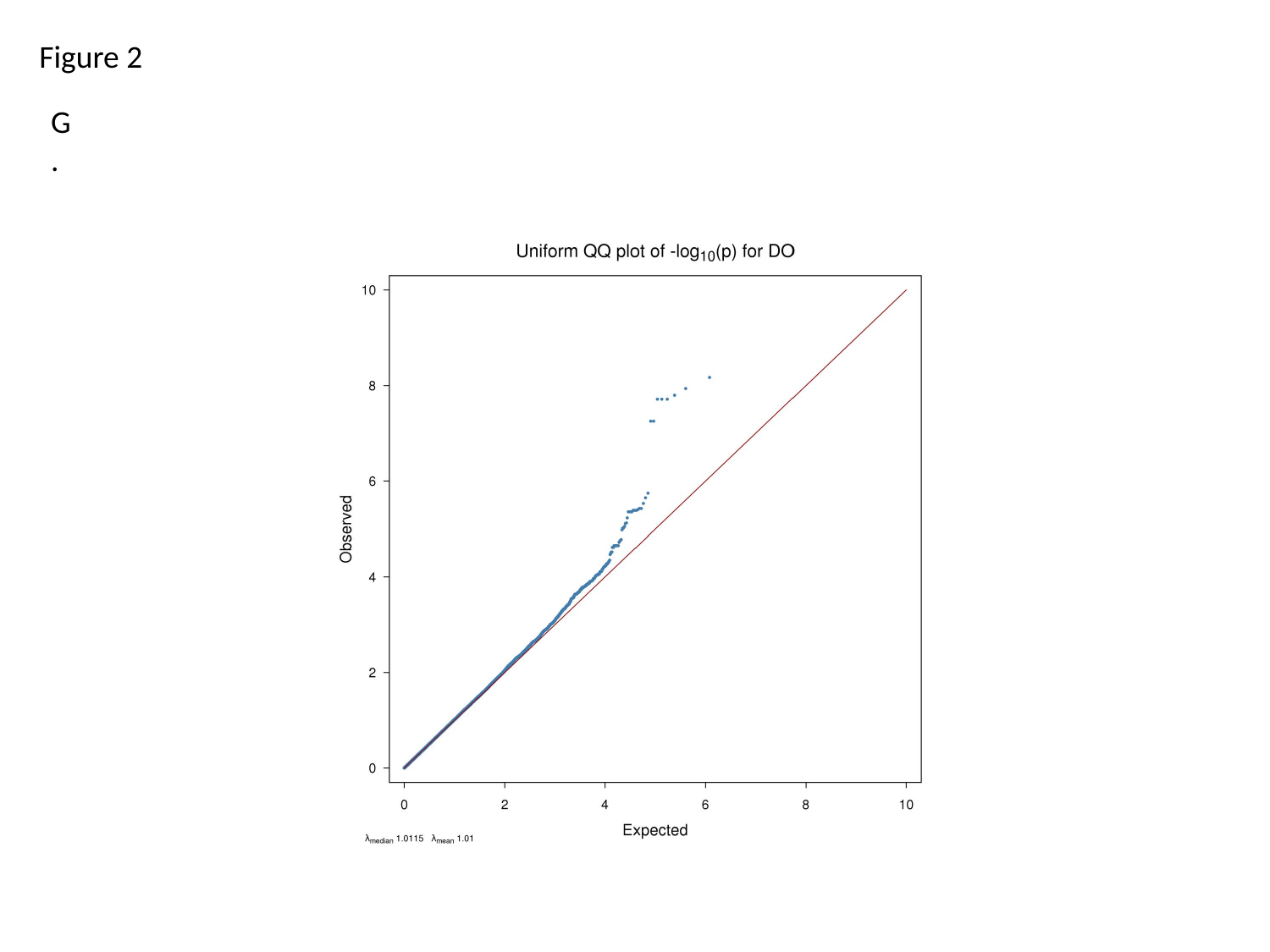

Figure 2
G.
